# An Observational Study of Material Durability of Three World Health Organization–Recommended Long-Lasting Insecticidal Nets in Eastern Chad

**DOI:** 10.4269/ajtmh.2012.11-0331

**Published:** 2012-09-05

**Authors:** Richard Allan, Laura O'Reilly, Valery Gilbos, Albert Kilian

**Affiliations:** The MENTOR Initiative, Crawley, United Kingdom; The MENTOR Initiative, Goré, Chad; Institut de la Francophonie pour la Medecine Tropicale, Vientiane, Laos; Malaria Consortium, London, United Kingdom

## Abstract

A total of 876 nets (229 Interceptor^®^, 363 Olyset^®^, and 284 PermaNet^®^) were collected 14 months post-distribution of long-lasting insecticidal nets (LLINs) from 811 households of internally displaced and host communities in Dar Sila District in eastern Chad to examine their physical condition. Holes were recorded by using three hole categories (average diameter = 2, 3.5, and 15 cm) and a Proportionate Hole Index (pHI). A total of 69.5% were in poor or very poor condition. There was no significant difference in the performance between the polyester 75 denier LLINs, but they had 4.22 times the odds of having a pHI ≥ 175 (poor or very poor condition) than polyethylene 150 denier LLINs; and 39.2% were unserviceable (pHI ≥ 300) compared with only 7.7% of the polyethylene LLINs. These results provide the first comparative data on LLIN material durability to guide procurement and replacement practice, and to inform urgently needed changes in LLIN international minimum specifications and product standards.

## Introduction

The international public health community is currently focused on achieving the Millennium Development Goals; reducing infant and child mortality by two-thirds by 2015 is a central goal. Malaria is the single greatest contributor of mortality in children less than five years of age across sub-Saharan Africa. Reducing malaria transmission and sustaining that reduction relies primarily on achieving universal coverage and use long-lasting insecticidal nets (LLINs).^1^ In 2009, the estimated investment in this intervention was $2.0 billion, and there is an estimated additional demand of $1.7 billion annually until 2015.^2^

Long-lasting insecticidal net technology is based on the slow release of pyrethroid insecticides, rendering them wash-resistant and extending insecticide residual effectiveness to at least three years without the need of re-treatment;^3^ its operational advantages are clear. However, a critical question, which has direct bearing on the success of malaria prevention and control, remains: how long does an LLIN last, in serviceable condition, under real life conditions? Current budgeting for LLIN distributions and subsequent replacement needs are based on an assumption that LLINs have an average useful life of 3–5 years.^4^ However, there is a significant lack of research in LLIN survival time, the variation in performance between LLINs of different textiles, and the general environment in which the net is being used (climate, housing, sleeping place, and washing patterns). Acknowledging this deficit, the Vector Control Working Group of the Roll Back Malaria (RBM) Partnership has created a work stream that calls for more field research on LLIN durability.^5^

There are two principal method challenges in measuring the serviceable life of an LLIN. The first is to determine the physical condition of the LLIN after long periods because many of the nets have been discarded. The second is to agree on a single, composite measure that quantifies the amount of damage to LLINs to enable comparison between different LLIN types.^6^

We present results of a cross-sectional comparison of the physical condition of three of the most widely used LLINs recommended by the World Health Organization Pesticide Evaluation Scheme under large-scale field use conditions. Two were a 75 denier (D) (filament mass per defined length) polyester-based LLIN, and the other was a 150D polyethylene-based LLIN. The condition of the LLIN was assessed after an average of 14 months use in Chad by applying an improved Proportionate Hole Index (pHI) as the outcome measure building on previously published work.^7^

## Methods

### Study area and population.

Dar Sila district is located in the Sahelian region of eastern Chad bordering Darfur, Sudan. It is characterized by its harsh semi-arid environment and a history of insecurity. It has been host to 35,000 Sudanese refugees since 2004 and more than 105,000 internally displaced people (IDP) since 2007. Mass population displacement and limited access to health care increased susceptibility to malaria; as a result, 23% of all admissions to the district's health system were because of malaria (United Nations High Commission for Refugees, Chad, unpublished data). Malaria transmission is seasonal and peaks during and at the end of the rainy season (June–November).^8^ The main vectors of malaria in this region are *Anopheles arabiensis* and *An*. *funestus*, and *An. coustani*, *An. nili*, and *An. pharoensis* are reported occasionally.^9^

In 2007–2008, 58,658 LLINs were distributed to host (10,588 Interceptor^®^ [BASF, Ludwigshafen, Germany], 993 PermaNet^®^ [Vestergaard Fransden, Lausanne, Switzerland], and 15,313 Olyset^®^ [Sumitomo Chemical Co., Tokyo, Japan]) and IDP (17,222 Interceptor^®^, 3,346 PermaNet^®^, and 11,196 Olyset^®^) communities across Dar Sila as part of a universal district-wide campaign. Interceptor^®^ and PermaNet^®^ LLINs were polyester 75D (PET-75D), and Olyset^®^ LLIN were polyethylene 150D (PE-150D). The LLINs were marked with identification numbers and distributed to all households. All households received malaria prevention education to guide correct use. In the following week, each household was visited by a team member who ensured that all LLINs were hung above sleeping areas, thereby maximizing correct use.

### Study design and sample size.

A cross-sectional household study was conducted in January 2009 during the dry season. A two-stage cluster sampling method was used to randomly select households from those that had received an LLIN during the 2007–2008 distribution campaign.

Sample size was calculated by using the standard formula for estimating a population proportion, n = (Z/d)^2^ × P(1 – P) multiplied by the design effect (2.6) assuming a loss-to-follow-up of 10% and a specified absolute precision of 8%. Two study groups (host and IDP) of 429 households each were required. From each study group, 44 clusters were randomly selected by using the probability proportional to size method. Simple random sampling was used to select 10 households from each cluster to give an overall sample size of 880. Households without an LLIN were excluded because the study was interested in LLIN material condition, not coverage.

### Field procedures.

Households sampled for the study were visited so that householders could be interviewed and the condition of LLINs assessed. Use of LLINs was determined by asking which individuals slept under it the previous night. Socioeconomic status (SES) was assessed by using an index based on household assets and housing conditions obtained by principal component analysis and then dividing the sample into wealth quintiles. Household measures included type of sleeping surface (mattress or floor/mat) and wall material. Each LLIN sampled was mounted onto a 180 cm × 160 cm wooden frame marked with horizontal bands 10 cm apart. The size of holes, divided into ≤ 2 cm, > 2–5 cm, and > 5 cm diameter categories; their position on the LLIN; and the quantity of holes in each category were recorded. Hole categories were designed to be easily and accurately measured under field conditions. The approximate sum of the areas of the holes was calculated for each size category based on a mean hole diameter of 2, 3.5, and 15 cm for the three hole categories, respectively. Categories were weighted 1, 3 and 56, respectively.

The number of holes in each category was multiplied by the category weight and expressed as a pHI. This approach has recently been adopted in a slightly modified version by the WHO guidelines for the monitoring of LLIN durability in the field^10^ after its development and use in various previously published studies.^11^ Although there is no consensus on at what point LLIN damage negates insecticide effect, this index is an attempt to standardize LLIN damage. This procedure, alongside insecticide bioassays, provides an overall assessment of net durability. The pHI was categorized into 4 groups (0–24, 25–174, 175–299, and ≥ 300) in which a pHI of 24 is equivalent to a total hole surface of 100 cm^2^.This value corresponds to having no hole in the >5 cm diameter category and no more than 8 holes in the > 2–5 cm diameter category, in the absence of any other holes, and is considered to be a serviceable LLIN.^11^ Previous work indicates that PET-75D are associated with less regular use once pHI > 25, indicating that users perceive nets to be of no use after this point despite continuing insecticidal activity. The highest category of a pHI ≥ 300 is equivalent to a total mean hole surface of 1,000 cm^2^ (i.e., a hole surface 10× greater than that with a pHI of 24), corresponding to ≥ 5 large holes. Based on the scarce literature available to date, these nets can be considered seriously damaged and of questionable benefit to the user.^12^

### Data handling and statistical analysis.

Data were double-entered independently into EpiData version 3.1 (The EpiData Association, Odense, Denmark), cleaned and then analyzed by using STATA version 11.0 (StataCorp LP, College Station, TX). For socioeconomic quintiles, a wealth index was calculated based on households assets by using principal component analysis.^13^ Wealth quintiles were calculated separately for the host and IDP population to account for different distributions of the wealth index in these two domains. Univariate analysis was performed against known potential explicatory variables by using two outcome measures: the pHI as a continuous variable comparing the mean between groups of interest, and the grouped pHI as described above. Multivariate analysis was performed to assess predictors of physical condition. The dependent variable pHI was grouped as a binary outcome as either ≥ 175 or < 175; this is equivalent to a comparison between good to fair and poor to very poor condition. *P* values < 0.05 were considered statistically significant, and 95% confidence intervals were used throughout taking into account the cluster design. All analytical procedures took account of the multi-stage sampling design by using the svy family of commands in STATA and applying the sampling weights.

### Ethical considerations.

This observational study was planned with and approved by the regional Ministry of Health. Community leaders were informed before the study and all gave verbal consent before initiation. Written consent was then obtained on the day of the study from all participating households.

## Results

### Household characteristics.

The survey included 811 randomly selected households containing 876 LLINs (4 of the original 880 LLINs were excluded because of incomplete data for key variables): 229 Interceptors^®^, 363 Olyset^®^, and 284 PermaNet^®^. This sampling corresponded to 0.6% of Interceptors^®^originally distributed, and 1.4% and 2.4% of Olyset^®^ and Permanet^®^, respectively. The total population covered was 4,191, the mean number of persons per household was 5.2 (range =1–12), and there was an average of 1.1 children less than five years of age. The education level of respondents was generally low; only 6.7% of household heads had attended at least primary school. Only 1.7% (n = 7) of IDP respondents ever attended school compared with 11.5% (n = 47) of the host community respondents.

The socioeconomic status also differed significantly between hosts and IDPs; 23.4% (n = 94) of IDP households were in the lowest quintile compared with 5.4% (n = 22) of host households, and 3.2% (n = 13) of IDP households were in the highest quintile compared with 37.4% (n = 153) of host households. The average number of LLINs per household was 1.1 (range = 1–3 LLINs in IDP and host communities). Consequently, the ratio of the mean number of LLINs to household residents was 1.1:5.2 or 1 LLIN for every 4.7 persons. When interviewed, 57.7% (n = 2,418) of household members reported to have slept under an LLIN the previous night and in 31.2% (n = 253) of the households, all members claimed to have slept under an LLIN the previous night.

Age of LLINs (length of household ownership) varied between 11 and 23 months. The average age of Interceptor^®^ was 12 months compared with 15 months for Olyset^®^ and Permanet^®^. Despite this difference, there was no evidence that age of LLINs had any significant impact on physical condition. Therefore, LLIN age was omitted from further analysis.

### Physical condition of LLINs.

Of the total LLINs observed, 25% (n = 219) had a pHI ≥ 300 after an average use of one year and were considered unserviceable and irreparable. A total of 44.5% (n = 390) were in the middle pHI categories (25–174 and 175–299), implying that these LLINs were only in partial service. Only 30.5% (n = 267) of LLINs had a pHI < 25 and were considered to be in good condition.

The proportion of Interceptor^®^ and PermaNet^®^ LLINs with a pHI ≥ 175 was 60.0% and 63.3%, respectively (*P* = 0.6). The difference was not significant and because these two brands are both PET-75D, they were combined and LLIN performance was compared with respect to the fiber characteristic (material and denier, i.e., PE-150D and PET-75D) instead of LLIN brand.

Of all LLINs, 81.7% were reportedly used only for sleeping. Of these nets, 59.0% were PET-75D and 41.0% PE-150D; they had a mean pHI of 209. The remaining 18.3% LLINs, as well as being used for sleeping, were used for fishing, of which 82.1% were PET-75D and 17.9% were PE-150D (*P* = 0.0001). These nets were in poor condition and had a mean pHI of 463.

**Figure 1.**
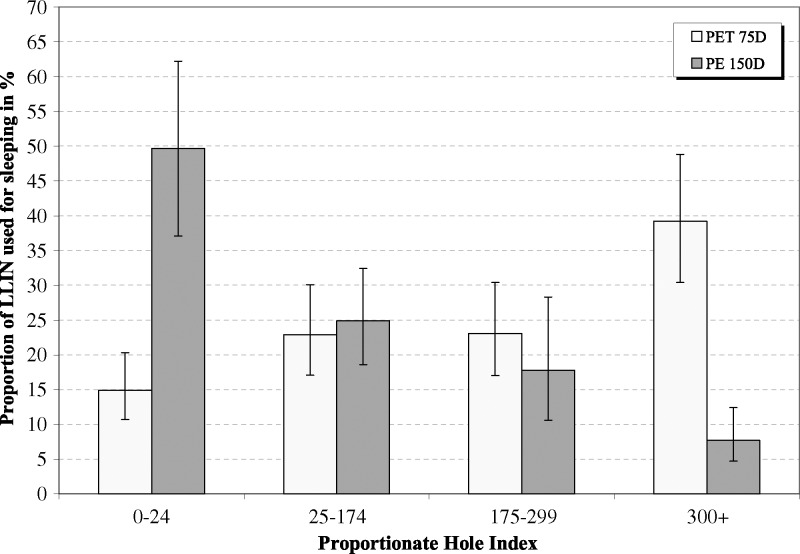
Performance profile of LLIN (used only for sleeping under) in Chad represented by the proportionate Hole Index (pHI) and according to material type: PET-75D and PE-150D. Error bars indicate mean ± SD.

There was a significant difference in performance between PET-75D and PE-150D LLINs. The LLINs used for purposes other than sleeping were excluded. Figure 1 shows a reversed performance profile of PE-150D versus PET-75D LLIN. A significantly greater proportion of PET-75D LLINs were found to be severely damaged, i.e., pHI ≥ 300 (*P* < 0.001).

### Regression analysis.

Stepwise logistic regression analysis was conducted to identify significant predictors for development of holes in LLINs used for sleeping. The number of residents per household and LLIN age were not significant and were excluded from the final model. In addition, although construction material of house walls (mud or brick) was significant on its own, once sleeping place was controlled for, construction material of house walls became insignificant and therefore was also excluded from the final model.

Five factors were significant predictors for LLIN physical condition or had at least borderline significance Table 1, and the variable identifying the two study domains (host or IDP population) was also included to reflect the sampling design. In the final model, the strongest association was between different LLIN fibers (material and denier); PET-75D had 4.22 greater odds of having a pHI ≥ 175 than PE-150D. Poorer sleeping conditions also proved a highly significant predictor of poor net condition, i.e., having a pHI ≥ 175 had an odds ratio of 3.10 (*P* < 0.0005). The LLINs used for fishing and sleeping had 2.18 (*P* = 0.019) times the odds of having a pHI ≥ 175 compared with LLINs used only for sleeping. Households in the three poorest wealth quintiles (SES = 1–3) had 1.52 (*P* = 0.057) times greater odds of having a pHI ≥ 175 compared with households with an SES = 4–5.

Surprisingly, results show that if there was one child less five years of age in the household, LLINs had 1.58 (*P* = 0.027) times greater odds of having a pHI ≥ 175 compared with having no children or more than one child. Table 2 shows the logistic regression model as described above but omits the non-significant variables described in the earlier model. Overall results did not change dramatically, but the difference between polyethylene and polyester LLINs increased slightly, giving an odds ratio of 4.75. Also, the odds ratio and significance levels increased slightly for nets used for fishing and households from the lowest three wealth quintiles. Finally, an alternate model using the five significant explanatory variables and sampling domain was run using the continuous measure of the pHI as the independent variable Table 3. The strength of the associations remained similar compared with the model with the dichotomous outcome variable (pHI > 175), except that wealth quintiles were no longer significantly associated with increasing hole index in the continuous model. Instead, the domain (host population or IDP) now was statistically significant suggesting some collinearity between these two variables.

## Discussion

The study results show that of the total LLINs observed, less than one-third (30.5%) were in serviceable condition after an average of 14 months use in the semi-arid conditions of eastern Chad. The results also show that 39.2% of PET-75D and 7.7% of PE-150D were badly torn and considered unserviceable (Figure 1). If one considers that the study did not include houses that no longer had LLINs, the level of damage may in fact be greater than that presented here. Households without nets were likely to have discarded them because of severe damage. This has been confirmed by follow-up studies that asked respondents why LLINs were missing. Within this short time frame, PET-75D LLIN had 4.22 times greater odds of having a pHI ≥ 175 compared with PE-150D LLINs. The PET-75D and PE-150D displayed reverse performance pHI profiles when used under these field conditions. After LLIN material type and denier, sleeping without a mattress was the next significant factor likely to further reduce the serviceable life of an LLIN. Although it would be of interest to estimate the average useful life of these products under the conditions of eastern Chad, this is not possible with sufficient reliability based on our data. As outlined by the WHO guideline on monitoring of LLIN durability,^10^ the attrition rate, i.e., nets lost because of damage or other reasons, is needed in addition to the physical condition of the nets and this is not possible based on the applied sampling approach.

The PET multifilament yarns and PE monofilament yarns are the preferred materials for LLIN manufacturers because of wide availability and relatively low cost of raw materials and the material characteristics of the finished LLINs. In 2001 RBM/WHO set minimum specifications for LLINs made with these materials with the aim of promoting good quality products from manufacturers and guiding institutional buyers.^14^ These minimum specifications were updated in 2005.^15^ The RBM/WHO minimum specification of PET LLINs is a multifilament yarn with a minimum of 36 filaments and PE LLINs with a minimum yarn of 100D monofilament. Consequently, of LLINs tested and recommended by WHO (December 2010) six are PET-75D or 100D, one is PE-110D, and two are PE-150D. Because of the price advantage of the 75D, it is this LLIN that is most often procured.

Under laboratory conditions, the strength of PET and PE yarns varies according to denier and fiber diameter.^16^ Tenacity value describes the stress at which an individual filament breaks and, because it is measured in grams per denier, it eliminates the effects of different filament size and allows one filament to be directly compared against another. Commercially available PET and PE yarn densities, uses, and tenacity values vary greatly from those used in standard clothing to those in bullet-proof vests. Of those commonly used for clothing and LLIN, a single PET filament normally has up to double the tenacity value of a single PE filament of equal denier.^17^ However, theoretically each filament of PET multifilament yarn essentially behaves mechanically as an independent entity.^18^ A 75D yarn constructed of multiple filaments (even with as few as 25 filaments) should have a lower tenacity value than a monofilament 75D yarn.

This study provides convincing evidence that in real life use conditions in semi-arid eastern Chad, multifilament PET-75D LLINs are significantly weaker than monofilament PE-150D LLINs to such an extent that they show a marked reverse performance profile in the size and number of holes that develop. Although these PE-150D LLINs are significantly more durable than PET-75D LLINs, even these are not adequately durable to provide reliable protection over a minimum of three years in this setting. The reverse performance profiles of these different LLIN types indicate that LLIN construction specifications largely determine the size and number of holes that develop in LLINs over time under real life use conditions.

Studies conducted in tropical environments in Africa have only assessed durability of LLINs of the same type; comparison of the performance of LLINs of different types is absent. Two such studies were conducted in Liberia and Ghana. In Liberia, 70% of Interceptor^®^ PET-75D LLINs (n = 383) had no holes after 12 months field use,^19^ indicating that LLINs may last better in a non-arid environment. However, in Ghana, 49 PET-75D LLINs recovered after 38 months use had an average of 40.5 holes > 0.5 cm per net and 50% of them had seam failures.^20^ Unfortunately, these publications do not give sufficient detail to enable calculation of an approximate pHI for a direct comparison.

Because RBM/WHO have set minimum specifications of denier for PET LLINs and PE LLINs, it is unsurprising that most international procurement agencies, donors, ministries of health, and non-government organizations use them as the basic specifications for LLIN procurement. Decisions on LLIN procurement are primarily made on the basis of cost. Thus, procurement agencies are deterred from requesting, and manufacturers and suppliers are deterred from offering, improved specification and ultimately more expensive LLINs. Production of PET-75D LLINs is faster and cheaper than production of PE-LLINs,^18^ thereby giving it a competitive advantage over PE LLINs, and resulting in an uneven marketplace with a prevalence of inadequate LLINs regarding durability and serviceable life.

The RBM/WHO minimum specifications for PET and PE LLIN need to be significantly increased for semi-arid settings; also further research on PET and PE yarn “equivalence” (denier and filament number per yarn) needs to be conducted if increased durability of LLINs and improved sustainability of malaria control are to be achieved. However, it is clear that significantly increasing the denier and tenacity value of PET and PE LLINs will increase manufacturers' costs and reduce production rates, resulting in more expensive LLINs. Donors will need to commit to meeting this increased initial cost in return for longer LLIN serviceable life and greater overall cost efficacy (i.e., cost per month of malaria protection gained per person).

International LLIN budgeting for procurement and replacement planning is currently based on a speculated LLIN serviceable life of 3–5 years. An alternative solution to increasing dernier specifications would be to replace PET-75D nets more frequently than the current guidelines suggest; this replacement would have high associated costs and would not be operationally and financially feasible in many settings. Reducing infant and child mortality by two-thirds by 2015 is a key millennium development goal. One of the means by which international donor and aid communities are attempting to achieve this goal is by large-scale provision of LLINs to malaria-endemic countries. However, there is a serious risk that achieving lasting health gains in a large proportion of malaria-endemic countries will fail because of a dependence upon LLINs of inadequate durability.

Planned international expenditure on LLINs post 2015 is expected to decrease significantly if international governments perceive that adequate numbers of LLINs to achieve universal coverage have been procured. Public health history shows that governments are likely to redirect their budgets to other health targets before measuring the sustainability of malaria health gains with current tools. Therefore, urgent action to improve significantly the material durability of LLINs for semi-arid settings is needed if the current large-scale investment is not to be wasted, and the opportunity to achieve sustainable malaria control gains is not to be lost.

## Figures and Tables

**Table 1:** Significant variables (*P* < 0.05) included in final multivariate logistic regression model for LLINs caused by a significant association with pHI ≥ 175, Chad*

Variable	Odds ratio (95% CI)	*P*
Material of LLIN		
PE-150D	1.0	
PET-75D	4.22 (2.37–7.86)	< 0.0005
Sleeping place		
Mattress	1.0	
Floor/mat	3.10 (1.92–5.03)	< 0.0005
Net use		
Only sleeping	1.0	
Fishing and sleeping	2.18 (1.14–4.16)	0.019
Socioeconomic status		
Higher quintiles (4–5)	1.0	
Lower quintiles (1–3)	1.52 (0.99–2.34)	0.07
Children < 5 years of age		
None or > 2 children	1.0	
One child	1.58 (1.06–2.38)	0.027
Sampling domain		
Host population	1.0	
IDP	1.55 (0.89–2.67)	0.11

* LLINs = long-lasting insecticidal nets; pHI = Proportionate Hole Index; CI = confidence interval; PE = polyethylene; PET = polyester; IDP = internally displaced people.

**Table 2:** Alternative model for association of LLINs with pHI ≥ 175 with core variables but leaving out significant variables in the model, Chad*

Variable	Odds ratio (95% CI)	*P*
Material of LLIN		
PE-150D	1.0	
PE-150D	4.75 (2.40–9.38)	< 0.0005
Sleeping place		
Mattress	1.0	
Floor/mat	3.09 (1.90–5.02)	< 0.0005
Net use		
Only sleeping	1.0	
Fishing and sleeping	2.45 (1.36–4.42)	0.003
Socioeconomic status		
Higher quintiles (4–5)	1.0	
Lower quintiles (1–3)	1.60 (1.03–2.47)	0.036
Children < 5 years of age		
None or > 2 children	1.0	
One child	1.64 (1.08–2.49)	0.021
Sampling domain		
Host population	1.0	
IDP	1.67 (0.90–3.10)	0.10
Age of net, months		
10–12	1.0	
13–17	1.39 (0.64–3.00)	0.40
18–23	1.14 (0.51–2.55)	0.75
Persons in household		
1–3	1.0	
5–6	1.05 (0.60–1.83)	0.86
7–12	1.08 (0.53–2.21)	0.82
Material of walls		
Mud or thatch	1.0	
Brick	1.20 (0.56–2.57)	0.63

* LLINs = long-lasting insecticidal nets; pHI = Proportionate Hole Index; CI = confidence interval; PE = polyethylene; PET = polyester; IDP = internally displaced people.

**Table 3:** Variables included in final multivariate logistic regression model for LLINs but using continuous pHI measure as independent variable, Chad*

Variable	Coefficient (95% CI)	*P*
Material of LLIN		
PE-150D	1.0	
PET-75D	230.0 (149.6–311.3)	< 0.0005
Sleeping place		
Mattress	1.0	
Floor/mat	172.9 (89.3–256.6)	< 0.0005
Net use		
Only sleeping	1.0	
Fishing and sleeping	184.1 (34.5–333.7)	0.017
Socioeconomic status		
Higher quintiles (3–5)	1.0	
Lower quintiles (1–2)	–4.9 (–80.3 to 70.5)	0.89
Children < 5 years of age		
None or > 2 children	1.0	
One child	132.8 (19.5–159.2)	0.022
Sampling domain		
Host population	1.0	
IDP	86.9 (13.2–159.2)	0.021

* LLINs = long-lasting insecticidal nets; pHI = Proportionate Hole Index; CI = confidence interval; PE = polyethylene; PET = polyester; IDP = internally displaced people.
